# Nanocrystalline Cellulose-Supported Iron Oxide Composite Materials for High-Performance Lithium-Ion Batteries

**DOI:** 10.3390/polym16050691

**Published:** 2024-03-02

**Authors:** Quang Nhat Tran, Chan Ho Park, Thi Hoa Le

**Affiliations:** Department of Chemical and Biological Engineering, Gachon University, 1342 Seongnam-daero, Sujeong-gu, Seongnam-si 13120, Republic of Korea; tran.nhat147@gachon.ac.kr

**Keywords:** nanocrystalline cellulose, lithium-ion batteries, iron oxide, high electrochemical performance material, energy storage

## Abstract

Nanocrystalline cellulose (NCC) can be converted into carbon materials for the fabrication of lithium-ion batteries (LIBs) as well as serve as a substrate for the incorporation of transition metal oxides (TMOs) to restrain the volume expansion, one of the most significant challenges of TMO-based LIBs. To improve the electrochemical performance and enhance the longer cycling stability of LIBs, a nanocrystalline cellulose-supported iron oxide (Fe_2_O_3_) composite (denoted as NCC–Fe_2_O_3_) is synthesized and utilized as electrodes in LIBs. The obtained NCC–Fe_2_O_3_ electrode exhibited stable cycling performance, better capacity, and high-rate capacity, and delivered a specific discharge capacity of 576.70 mAh g^−1^ at 100 mA g^−1^ after 1000 cycles. Moreover, the NCC–Fe_2_O_3_ electrode was restored and showed an upward trend of capacity after working at high current densities, indicating the fabricated composite is a promising approach to designing next-generation high-energy density lithium-ion batteries.

## 1. Introduction

Transition metal oxides (TMOs) (e.g., MnO_2_, ZnO, Fe_2_O_3_, Fe_3_O_4_, and SnO_2_) have become mainstream electrode materials in the field of lithium-ion batteries (LIBs) due to their high theoretical capacity [1,2,3,4]. Among them, iron oxide (Fe_2_O_3_) has been attractive and extensively considered as an ideal alternative electrode material because of its high theoretical capacity (1007 mAh g^−1^), low conversion voltage, and good stability as well as environmental friendliness [5,6]. Unfortunately, poor cycling stability due to poor electrical conductivity and structural collapse as a result of large volume expansion during the lithiation/delithiation process results in poor rate performance and a short life of LIBs.

One of the efficient approaches to overcome the disadvantages of Fe_2_O_3_ is a composite of nanosized Fe_2_O_3_ particles and conductive carbon-based materials such as graphene and carbon nanotubes (CNT) [7,8,9,10,11,12,13,14]. Incorporating nanosized Fe_2_O_3_ particles with carbon-based materials could couple the advantages of both nano entities, which improve the contact between electrode and electrolyte, and conductive carbonaceous materials, which alleviate the volume change and nanosized particle aggregation [3,15,16,17,18]. Furthermore, including a composite of iron oxide particles into carbonaceous materials could be easily carried out through thermal annealing of organic matter and inorganic compounds [3,9,19,20,21,22,23].

Nanocrystalline cellulose (NCC), one of the green carbon sources for electrode composite materials, exhibits a negative surface charge in solution [24,25]. The high surface energy of metal oxide nanoparticles leads to their self-aggregation, and the NCC owns its negative surface charge in solution. Thus, the complexation of NCC to metal oxides will help to decrease the high surface energy of nanosized metal oxide particles, which effectively prevents the aggregation of nanosized metal oxide particles [26,27,28]. In addition, the large surface and crystalline structure of nanocrystalline cellulose not only serve as the structural template in the carbonaceous metal oxides composite synthesis process but also effectively confine the Fe_2_O_3_ nanosized particles in a restricted area, reducing the volume expansion during the charge/discharge process, which improves both electrochemical performance and the stability of the electrode materials during the cycling of LIBs [27,28,29,30,31,32,33]. In addition, NCC contains numerous hydroxyl groups on its surface. Through various chemical modification processes, such as oxidation of the primary hydroxyl groups at the C6 position, carboxyl groups (-COOH) can be introduced onto the NCC surface (Figure S1) [34,35]. These carboxylic groups can interact with lithium ions and electrolyte components during battery operation and can facilitate the formation of a stable SEI layer by participating in the passivation reactions and stabilizing the solid–electrolyte interface. In addition, surface chemical modification and functionalized nanocrystalline cellulose using sodium periodate oxidation will transform at least part of the carboxylic groups of nanocrystalline cellulose into sodium carboxylate (-COO- +Na) groups. The sodium carboxylate groups in nanocrystalline cellulose materials could dissociate sodium ions into electrolytes, which can enhance the stable solid electrolyte interphase (SEI) layer formation and improve the stability of LIBs in long-term performance [35,36,37,38,39,40,41,42]. Furthermore, NCC’s electrochemical stability, mechanical strength, and natural sustainability also enhance the efficiency in composite synthesis and structural maintenance [26,27,43]. The low cost and light weight of NCC also help to extend its industrial and commercial applications, such as in anticorrosion coatings [44], single-molecule force spectroscopy (SMFS) measurements [45], electrochemical sensors [46], and in flexible energy and electronic devices [47].

In this work, we produced nanocrystalline cellulose-incorporated nanosized Fe_2_O_3_ particles to construct a composite as an electrode material for LIBs. The introduction of the NCC framework can effectively buffer the volume expansion and contraction during the discharge/charge process. The crystalline structure of NCC can promote lithium-ion transport and increase lithium storage [26,27,29,43]. The combination of nanosized Fe_2_O_3_ particles and NCC formed a high electrochemical performance composite, which delivers high specific capacity, outstandingly long cycle stability, and excellent rate performance when employed as an electrode material for LIBs.

## 2. Experiment Details

### 2.1. Materials

Spray-dried nanocrystalline cellulose powder derived from wood pulp, with dimensions of approximately 5 nanometers (nm) in diameter and 150–200 nanometers in length, purchased from Process Development Center, University of Maine, Orono, ME, USA (Lot #2015-FPL-CNC-071) is used as a cellulose source. Other chemicals, namely iron (II) chloride tetrahydrate (FeCl_2_·4H_2_O) and sodium citrate tribasic dihydrate (C_6_H_5_Na_3_O_7_·2H_2_O) were purchased from Sigma Aldrich Co., Ltd. (St. Louis, MO, USA) with all analytical grades and used without further purification.

### 2.2. Fabrication of Nanocrystalline Cellulose-Supported Iron Oxide Composite

The composite precursor was composited from mixtures of NCC, FeCl_2_·4H_2_O, and C_6_H_5_Na_3_O_7_·2H_2_O by using ethanol and deionized (DI) water (1:1) as a solvent. In the specific process, 50 nmol FeCl_2_·4H_2_O and 100 mmol C_6_H_5_Na_3_O_7_·2H_2_O were alternatively dispersed in 40 mL of an ethanol–water solution under vigorous stirring at room temperature for 1 h. A total of 1g of NCC was added into the solution and stirring continued at room temperature for 1 h to form a homogenous solution. The solution was then loaded into a 100 mL stainless steel autoclave. The solution was maintained for 8 h at 180 °C in an oven and then naturally cooled to room temperature. The precursor was collected via centrifugation, washed with DI water, and dried at 25 °C overnight. The obtained precursor was first stabilized for 4 h at 200 °C and then carbonized for 2 h at 500 °C in a nitrogen atmosphere with a heating rate of 5 °C min^−1^. The annealed sample that formed the nanocomposite structure with carbonized NCC and Fe_2_O_3_ was noted as NCC–Fe_2_O_3_. Then, NCC–Fe_2_O_3_ was designated as the electrode material for high-performance LIBs. A similar fabrication process was carried out under the same conditions without the existence of NCC and the final product was noted as Fe_2_O_3_ as a comparison electrode material.

### 2.3. Characterization

Powder X-ray diffraction (XRD) patterns were recorded on a diffractometer (Rigaku/Smartlab, Tokyo, Japan) at 40 kV, 30 mA X-ray generator with a Kβ filter for Cu radiation over the 2θ range of 10–100° at a scanning rate of 1.0° min^−1^. Thermogravimetric analysis (TGA) was performed under atmospheric conditions with a heating rate of 10 °C min^−1^. The morphologies and nanostructure of the samples were studied with a Hitachi S-4700 (Hitachi Ltd., Tokyo, Japan) field emission scanning electron microscope (SEM),with an accelerating voltage of 15kV, and a Tecnai F30S-Twin (Hillsboro, OR, USA) transmission electron microscope (TEM), with an accelerating voltage of 300 keV. The TEM observation samples were prepared by dropping sample-dispersed ethanol solution onto the copper grids. The elemental content and maps were obtained via energy-dispersive X-ray analysis (EDS). The size distribution of Fe_2_O_3_ was evaluated based on the SEM images. X-ray photoelectron spectroscopy (XPS) of the NCC–Fe_2_O_3_ composite was recorded on a PHI 5000 (ULVAC-PHI, Inc., Chigasaki, Kanagawa, Japan) to determine the element content of the composite, and Brunauer–Emmett–Teller (BET) specific surface areas of the NCC–Fe_2_O_3_ composite were determined via N_2_ adsorption at 77.3 K (Micromeritics, ASAP 2020, Norcross, GA, USA).

### 2.4. Electrochemical Performance Measurement

All the testing samples were prepared using a CR2023-type coin cell (Rotech Inc., Gwangju, Republic of Korea) in a glove box filled with pure argon to investigate the electrochemical performance of the NCC–Fe_2_O_3_ composite, and to compare the Fe_2_O_3_ sample. The electrode (mass load 0.88 mg/cm^2^) was composed of the obtained composites (70%), polyvinylidene fluoride (PVDF) binder (15%), and carbon black (15%), which were dissolved in an N-methyl pyrrolidone (NMP) solvent to form a smooth slurry. Then, the slurry was uniformly coated on a copper foil (r = 0.65 cm) and dried overnight at 70 °C in a vacuum oven for 24 h. Li foil was used as the anode, polyethylene membrane was applied as a separator, and 50 μL of 1 M LiPF_6_ in a mixed solvent of ethylene carbonate (EC)/dimethyl carbonate (DMC) with a volume ratio of 1:1 were used as an electrolyte for each electrode. Charge and discharge tests were conducted on a battery cycler (NanoCycler-01, NANOBASE, Geumcheon-gu, Seoul, Republic of Korea) over a potential range of 0.01~3.0 V vs. Li^+^/Li under a constant current density of 100 mA g^−1^ at room temperature. The rate performance tests were conducted on a range between 100 and 10,000 mA g^−1^ current densities. Cyclic voltammetry (CV) behaviors were studied by using a battery-cycle tester (WBCS3000, WonAtech, Seocho-gu, Seoul, Republic of Korea) with a voltage window of 0.01–3.0 V at a scan rate of 0.1 mV s^−1^. Electrochemical impedance spectroscopy (EIS) of the batteries was performed on the ZIVE MP1 (WonAtech, Seocho-gu, Seoul, Republic of Korea) analyzer, and the frequency was set from 100 kHz to 100 MHz with an AC amplitude of 10 mV.

## 3. Results and Discussion

**Figure 1:**
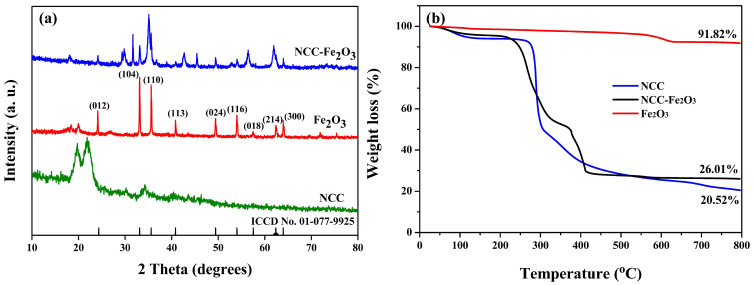
Typical characterizations of the obtained composite NCC–Fe_2_O_3_ and Fe_2_O_3_ sample. (**a**) XRD patterns and (**b**) TGA curves.

To determine the phase composition and physical properties of the materials, the NCC–Fe_2_O_3_ composite and comparison Fe_2_O_3_ sample were first identified with X-ray diffraction, and the obtained results are shown in Figure 1a. As seen in Figure 1a, the diffraction peaks at 2θ = 24.33, 33.127, 35.607, 40.826, 49.443, 54.018, 57.54, 62.378, and 63.963° for the Fe_2_O_3_ sample are highly indexed to the characteristic patterns of Fe_2_O_3_ [ICCD No. 01-077-9925], which correspond to the (012), (104), (110), (113), (024), (116), (018), (214), and (300) planes of Fe_2_O_3_. There are no impurity peaks, and all the diffraction peaks are sharp, suggesting high purity and well-crystallized Fe_2_O_3_. Meanwhile, all diffraction peaks for the NCC–Fe_2_O_3_ composite are consistent with the characteristic peaks of Fe_2_O_3_. Moreover, the weakened and no-shifted peaks of the composite illustrated that Fe_2_O_3_ particles had been well introduced into the composite and connected on the surface of NCC. In addition, the appearance of diffraction peaks at 2θ = 31.148, 35.612, 42.69, and 56.39° for NCC–Fe_2_O_3_, assigned to the amorphous state of carbonaceous materials [ICCD Card No. 01-046-0943], show a slight shift of the obtained peaks centered at 34 and 40.2°; the presence of sharp peaks designates the crystalline nature of NCC, while the comparison Fe_2_O_3_ sample does not display this diffraction peaks, indicating the presence of NCC in the NCC–Fe_2_O_3_ composite and that the NCC–Fe_2_O_3_ composite was successfully constructed. The characteristic peaks of Fe_2_O_3_ and sharp carbonaceous structure diffraction peaks can be found clearly in the composite XRD patterns not only indicating that Fe_2_O_3_ had been well embedded into the NCC network but also suggesting the presence of a nanocrystalline structure of NCC after thermal reaction, which will help to prevent the aggregation of nanosized Fe_2_O_3_ particles and enhance the electrochemical performance.

To analyze the content of NCC in the NCC–Fe_2_O_3_ composite, a thermal gravimetric analysis (TGA) was conducted from room temperature to 800 °C at a heating rate of 5 m^−1^, and the obtained results for the NCC–Fe_2_O_3_ composite, NCC, and Fe_2_O_3_ samples are shown in Figure 1b. According to the TGA curves, the NCC–Fe_2_O_3_ composite indicates a significant mass loss between 250 °C and 400 °C and maintains a constant weight above 400 °C, and the extant material weight is 26.01%. This is most likely attributed to the combustion of NCC in the NCC–Fe_2_O_3_ composite. In addition, the TGA curve of NCC shows a light mass loss of approximately 7–8% below 250 °C, corresponding to impurities and adsorbed water present in the air, which is consistent with the obtained result for NCC–Fe_2_O_3_, and the major weight loss displayed between 250 and 500 °C is considered to be related to the carbonization of NCC [48]. These results show that the carbonization of NCC can proceed at temperatures ranging from 250 to 500 °C and can be conducted simultaneously through thermal annealing of Fe_2_O_3_ nanoparticles into the carbonaceous structure during the composite synthesis. On the other hand, the Fe_2_O_3_ sample shows that there is not much change, with a weight loss of 8.18%, corresponding to adsorbed water, trace amounts of oxygen, and easily oxidizable matter in the sample. Based on the change in TGA curves, the content of NCC in the NCC–Fe_2_O_3_ composite can be calculated to be 65.81%.

**Figure 2:**
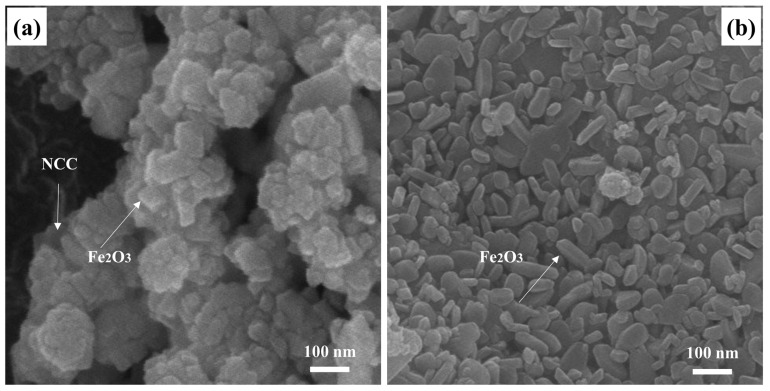
SEM images of (**a**) NCC–Fe_2_O_3_ composite and (**b**) Fe_2_O_3_ sample.

Figure 2 shows the typical morphologies of the NCC–Fe_2_O_3_ composite and a comparison Fe_2_O_3_ sample with the same magnification. It can be discerned that the NCC–Fe_2_O_3_ composite SEM image (Figure 2a) clearly shows that nanosized Fe_2_O_3_ particles are anchored on the NCC and exhibit an enlarged surface area, which is thus beneficial for enhancing the interface between materials and increasing the electrochemical properties. It is noted that the nanosized Fe_2_O_3_ particles almost retain the morphological properties in comparison to the typical morphological properties of the Fe_2_O_3_ sample, which can be obtained from Figure 2b. Furthermore, the majority of the nanosized Fe_2_O_3_ particles are exposed to the outside of the NCC, and a thin layer of NCC was coated on the surface of Fe_2_O_3_ that not only prevents the aggregation of Fe_2_O_3_, decreasing the volume expansion during the performance of composite, but also make sure that the Fe_2_O_3_ particles effectively connect with the lithium ions while avoiding direct contact with the electrolyte, exposing large electrochemical interaction sites and providing more active areas. In addition, three elements (Fe, C, and O) are explored in the composite, and the percentage of the three elements via EDS mapping are shown in Table 1. The percentage of Fe, C, and O are 14.98, 60.26, and 24.77%, respectively, which confirms the efficient assembly between Fe_2_O_3_ and NCC.

**Figure 3:**
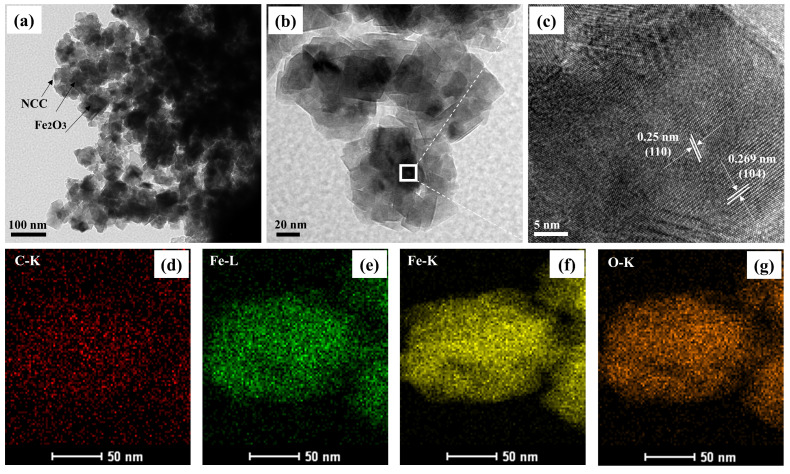
(**a**) Typical TEM image, (**b**,**c**) HRTEM images and EDS full elemental mapping images, and (**d**) C, (**e**,**f**) Fe, and (**g**) O of NCC–Fe_2_O_3_ composite.

At the same time, the further detailed morphology of the NCC–Fe_2_O_3_ composite was observed via TEM and high-resolution TEM (HRTEM), and the obtained images are shown in Figure 3. TEM images (Figure 3a,b) present the uniformity of the anchored Fe_2_O_3_ particles on the NCC network, and a thin layer of NCC can be noted on the TEM images. Although some nanoparticle aggregation is observed in Figure 3a, the amorphous and large surface can be seen from the TEM images with higher magnification (Figure 3b), indicating the hierarchical porous structure and homogeneous dispersion of Fe_2_O_3_, which contribute to electron transportation and alleviate volume change to enhance the structural stability during the cycle. The HRTEM of the NCC–Fe_2_O_3_ composite (Figure 3c) shows the internal high-resolution lattice fringes, which confirm the existence of crystalline Fe_2_O_3_ particles with the lattice parameter of 0.25 and 0.269 nm corresponding to the (110) and (104) crystal plane of Fe_2_O_3_, showing consistency with the XRD results. In addition, the corresponding elemental mapping images (Figure 3d–g) for the elements of C, Fe, and O annotate a homogeneous distribution of Fe_2_O_3_ onto the NCC, which verifies the successful synthesis of the NCC–Fe_2_O_3_ composite as per the prediction made in advance.

**Figure 4:**
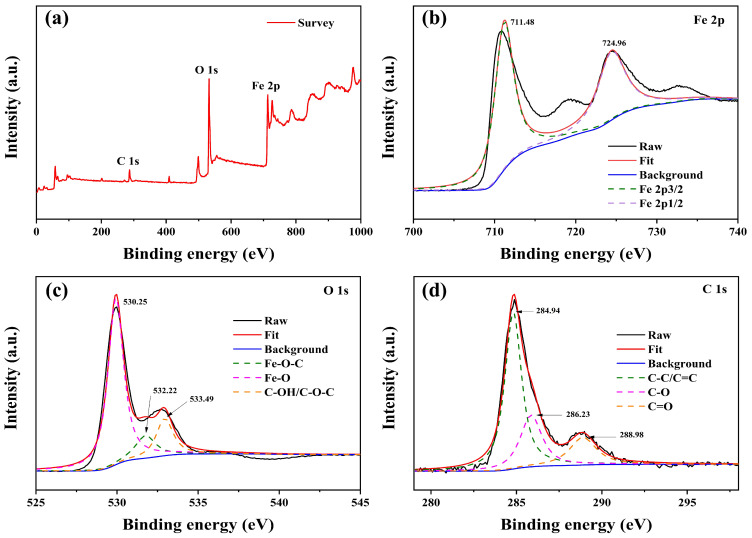
(**a**) XPS survey spectrum of NCC–Fe_2_O_3_ composite and high-resolution XPS peaks of (**b**) Fe 2p, (**c**) O 1s, and (**d**) C1s.

To further characterize the surface chemical composition of the NCC–Fe_2_O_3_ composite, X-ray photoelectron spectroscopy (XPS) was performed and the chemical element content and valence states are shown in Figure 4. According to Figure 4a, the full-range XPS survey spectrum consists of C 1s, O 1s, and Fe 2p with the peaks located at 287.05, 532.17, and 713.04 eV, respectively, which reveal that the NCC–Fe_2_O_3_ composite mainly contained three elements of Fe, C, and O.

In Figure 4b, the Fe 2p spectrum shows the two highest peaks at 711.48 and 724.96 eV, corresponding to Fe 2p_3/2_ and Fe 2p_1/2_, respectively, which further verify the presence of the Fe_2_O_3_ phase. A broad peak at 719.16 eV between Fe 2p_3/2_ and Fe 2p_1/2_ can be ascribed to the satellite peak of Fe^3+^ in Fe_2_O_3_, which is consistent with previous reports [11,20,49]. Figure 4c shows the detailed XPS spectrum of O 1s, which exhibits three peaks located at 530.25, 532.22, and 533.49 eV, demonstrating the Fe-O-C, Fe-O, and C-OH/C-O-C bonds to confirm the connection of Fe_2_O_3_ with NCC [10,20]. The peak positions of three parts divided in the C 1s XPS spectrum (Figure 4d) are 284.94, 286.23, and 288.98, corresponding to C-C/C=C, C-O, and C=O bonds, respectively, regarding the C atoms around the NCC [29]. The obtained XPS spectrums indicate that the NCC–Fe_2_O_3_ composite had been successfully fabricated.

**Figure 5:**
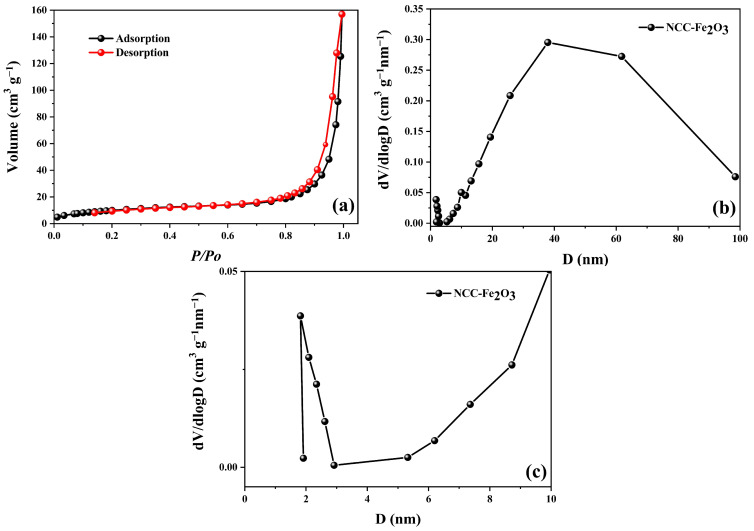
(**a**) Nitrogen adsorption–desorption isotherm, (**b**) BJH pore size distribution, and (**c**) nanopore distribution for the NCC–Fe_2_O_3_ composite.

The nitrogen isothermal adsorption and desorption of the NCC–Fe_2_O_3_ composite were measured to evaluate the surface areas and porous nature of the composite and the results are displayed in Figure 5. As shown in Figure 5a, the composite shows a typical IV characteristic isotherm, indicating the presence of abundant mesopores. The pore size distribution of the NCC–Fe_2_O_3_ composite is shown in Figure 5b and ranges from 2 to 98 nm with a maximum peak pore diameter centered at 40 nm, representing the mesopores and macropores included in the composite. In addition, Figure 5c also points out that nanopores with an average size of 4 nm also exist in the composite. Furthermore, the Brunauer–Emmett–Teller (BET) specific surface areas (SSA) and pore volume (PV) of the NCC–Fe_2_O_3_ composite are 53.79 m^2^ g^−1^ and 0.244 cm^3^ g^−1^, respectively. The large BET surface area and meso–macroporous features could establish enough open channels, which not only facilitate the transportation of ions but also shorten the Li^+^ diffusion pathways, leading to the better utilization of active materials and storage of lithium ions during the cycling process and improving the electrochemical performance of the composite [9,50,51]. Furthermore, nanopores appearing in the composite can act as buffering spaces to manage the volume change of Fe_2_O_3_ instead of being destroyed in the lithiation/delithiation process, which can preclude the volume expansion of Fe_2_O_3_ in the composite to enhance the cycling stability of LIBs.

**Figure 6:**
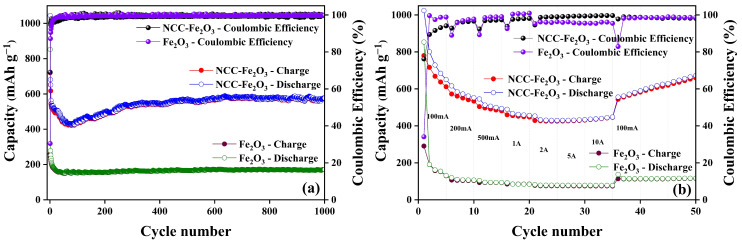
(**a**) Long-term cycling performance and coulombic efficiency at 100 mA g^−1^ and (**b**) rate capability performance of NCC–Fe_2_O_3_ composite and Fe_2_O_3_ sample.

According to the analysis results, the NCC–Fe_2_O_3_ composite, which exhibits excellent physical properties, and the comparison Fe_2_O_3_ sample were applied as electrode materials in LIBs to investigate their electrochemical performance. The cycling performance of NCC–Fe_2_O_3_ and Fe_2_O_3_ electrodes was carried out at 100 mA g^−1^ current density for 1000 cycles to compare the electrochemical properties of the obtained composite and comparison material. The long-term cycle charge/discharge capacities and coulombic efficiency of both electrode materials for LIBs are shown in Figure 6a. The NCC–Fe_2_O_3_ composite delivers initial discharge/charge capacities of 1044.35/720.61 mAh g^−1^ with a coulombic efficiency (CE) of 69%. However, the dramatic capacity decay of NCC–Fe_2_O_3_ can be seen in the first 5 cycles and the capacity slowly decreases in the following 100 cycles, exhibiting a capacity loss of 59%. The decrease in reversible capacity from 1044.35 to 433.12 mAh g^−1^ after 100 cycles was mainly attributed to the volume expansion and reconstruction of Fe_2_O_3_ [7,9,12,30]. After this stage, the capacity value shows a steady increase and can maintain and remain at approximately 576.70 mAh g^−1^ after the last 1000th cycle, remaining at 55% first capacity, which confirms the excellent cycling performance of the NCC–Fe_2_O_3_ composite electrode. In addition, the CE rises to more than 95% in the following few cycles and quickly stabilizes at ~100% in the subsequent 1000 cycles. The continuously increasing trend of charge/discharge capacity for the cycle performance test of Fe_2_O_3_-NCC LIBs would be explained with three aspects, including the activated process of Fe_2_O_3_ particles, meaning that some blocked sites could be gradually activated with the increase in testing cycles, the “pseudo-capacitance-type behaviors” which reduce down Fe_2_O_3_ particles size, and the extra lithium-ion storage based on higher electrochemical surface which can maintain a stable SEI layer [52,53]. On the other hand, the incorporated tolerant and flexible NCC can help the relatively smaller size Fe_2_O_3_ particles be more well dispersed and anchored on NCC frameworks to effectively increase the usage of active materials, resulting in an increase and higher capacity during the cyclability process. Furthermore, the carbonaceous support from NCC can not only accommodate a volume change during the charge/discharge process but also can compensate for the low conductivity of Fe_2_O_3_ particles.

Nevertheless, the specific charge/discharge capacities of the comparison Fe_2_O_3_ sample are only 852.35/259.84 mAh g^−1^ in the first cycle, drop dramatically to 189.52/183.61 mAh g^−1^ in between the 1st and 10th cycles, maintain an unchanged state during the cycling performance test, and remain at 171.35 mAh g^−1^ (20% of initial capacity) after 1000 cycles. The NCC–Fe_2_O_3_ composite not only delivers higher specific capacities than the Fe_2_O_3_ sample but also demonstrates excellent cycling stability with better capacity retention.

The rate capability of the obtained NCC–Fe_2_O_3_ composite and comparison Fe_2_O_3_ sample at the different current density ranges of 0.1, 0.2, 0.5, 1, 2, 5, and 10 A g^−1^ are displayed in Figure 6b. The NCC–Fe_2_O_3_ composite exhibits average discharge capacities of 777.78, 577.36, 511.84, 466.77, 435.38, 431.29, and 431.53 mAh g^−1^ for five cycles at 0.1, 0.2, 0.5, 1, 2, 5, and 10 A g^−1^, respectively, much higher than those of the comparison Fe_2_O_3_ sample, which only delivers average capacities of 298.58, 110.27, 96.29, 86.08, 79.56, 78.7, and 78.39 mAh g^−1^ for five cycles with the same conditions. Moreover, the NCC–Fe_2_O_3_ composite discharge capacity can recover to a high value of 556.07 mAh g^−1^ when the current density returns to 0.1 A g^−1^ and shows an upward trend of specific capacity for the next 15 cycles, confirming both the excellent rate performance as well as structural stability of the NCC–Fe_2_O_3_ composite. The favorable rate performance of the NCC–Fe_2_O_3_ composite can be allocated to the composite’s porous structure and the effective influence and supporting role of NCC [20]. The integration of nanosized Fe_2_O_3_ particles into the NCC with the porous large surface structure effectively enhances the lithium storage performance of the NCC–Fe_2_O_3_ composite, which emphasizes the synergistic effect of carbonaceous material and nanosized Fe_2_O_3_ particles. Furthermore, the flexible NCC not only provides a highly conductive network to shorten and ensure the dispersal of lithium ions but also serves as a supporting layer, which minimizes the volume expansion and prevents the aggregation of Fe_2_O_3_ particles upon cycling. Table 2 shows a summary of the improvement capabilities of the NCC–Fe_2_O_3_ composite and a comparison of the Fe_2_O_3_ sample electrodes. As can be observed, the NCC–Fe_2_O_3_ electrode shows better initial capacity and CE, as well as remaining capacity after 1000 cycles, which proves the better electrochemical performance of the NCC–Fe_2_O_3_ composite in comparison to the Fe_2_O_3_ sample.

**Figure 7:**
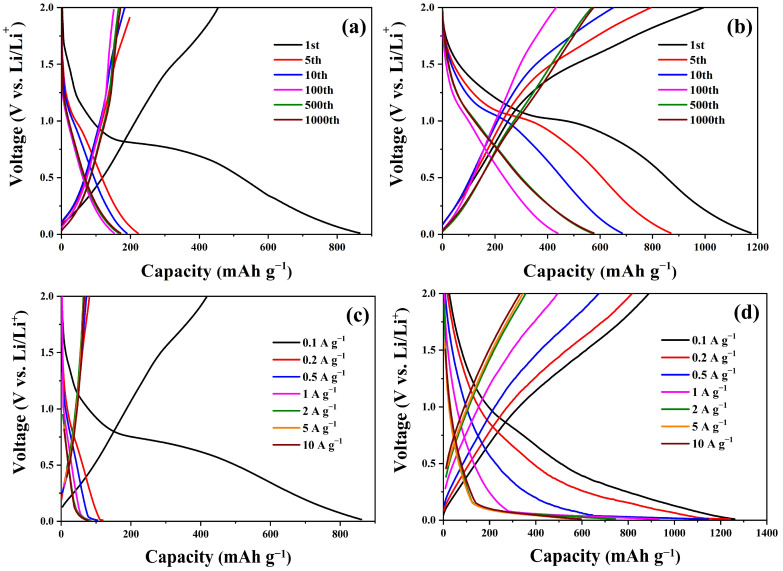
The galvanostatic charge and discharge profile of Fe_2_O_3_ sample and NCC–Fe_2_O_3_ composite (**a**,**b**) at 100 mA g^−1^ and (**c**,**d**) at various current densities.

The discharge/charge curves of the NCC–Fe_2_O_3_ composite and comparison Fe_2_O_3_ sample for the 1st, 5th, 10th, 100th, 500th, and 1000th cycles at a current density of 100 mA g^−1^ with a potential voltage from 0.01 to 2 V are displayed in Figure 7a,b. As for NCC–Fe_2_O_3_ (Figure 7b), the first discharge/charge ratio capacity of 1174.4/794.1 mAh g^−1^ leads to an initial CE for the first cycle of 67.6%. The low CE and capacity loss in the first cycle are a response to the formation of the solid electrolyte interphase (SEI) layer and the lack of activation of NCC/Fe_2_O_3_ [15,16,19]. The CE value steadily increased and reached approximately ~100% after five cycles, which can be the result of the interactions between the electrolyte and active materials. In addition, the discharge/charge specific capacities of NCC–Fe_2_O_3_ gradually decrease until the 100th cycle and exhibit an increasing trend and show a better capacity value within 500 and 1000 cycles, which indicates the good reversibility of NCC–Fe_2_O_3_ after the activation process. Following the formation of an SEI layer, the reduction of Fe_2_O_3_ can be proved by the plateau identified around 0.72 V at the initial cycle. However, the observed plateau disappeared gradually when the working cycle increased, and the similarly shaped curve of subsequent cycles almost overlapped between the 500th and 1000th cycle, indicating the enhancement of the NCC–Fe_2_O_3_ composite’s electrochemical stability and cyclability. In comparison, the Fe_2_O_3_ sample shows a large capacity loss in the first cycle with an initial CE for the first discharge/charge ratio capacity of 865.36/454.27 mAh g^−1^ of 52.49% and exhibits a significant decrease after the 1st cycle and remains at a discharge capacity of 171.25 mAh g^−1^, 19.79% of the initial discharge capacity, after 1000 cycles.

Figure 7c,d show the initial discharge–charge profiles of the NCC–Fe_2_O_3_ composite and the comparison with the Fe_2_O_3_ sample electrodes at various current rates. The first discharge capacities of the NCC–Fe_2_O_3_ composite were recorded around 1260.47, 1239.67, 1145.81, 932.91, 742.77, 577.78, and 595.95 mAh g^−1^, respectively, at 0.1, 0.2, 0.5, 1, 2, 5, and 10 A g^−1^. Meanwhile, the Fe_2_O_3_ sample delivers first discharge capacity values of 860.85, 119.44, 104.11, 84.63, 82.01, 74.94, and 74.95 mAh g^−1^, which are considerably lower than the NCC–Fe_2_O_3_ electrode, at the same current density. Those results indicate the better performance, even though working under high current densities, of the NCC–Fe_2_O_3_ composite as well as reconfirm the supporting role of NCC for achieving a good rate performance in the composite. In addition, the NCC–Fe_2_O_3_ discharge/charge curves did not show plateaus when low current densities were applied, while a clear plateau can be observed at around 0.72 V for the Fe_2_O_3_ sample at 100 mA g^−1^, which agrees with the results shown in Figure 6b. Moreover, a plateau below 0.25 V appears in both samples when the current densities are above 2 A g^−1^, indicating that the lithium storage process can be affected by electrochemical redox reactions at high current densities. In addition, the charge/discharge profiles of NCC–Fe_2_O_3_ show similar shape curves and higher, gradually decreased specific capacity at all current densities, which not only confirms that the NCC–Fe_2_O_3_ composite is more stable than the Fe_2_O_3_ sample in various current densities but also demonstrates the favorable maintenance of the electrode structural integrity and conversion reactions at diverse current densities.

**Figure 8:**
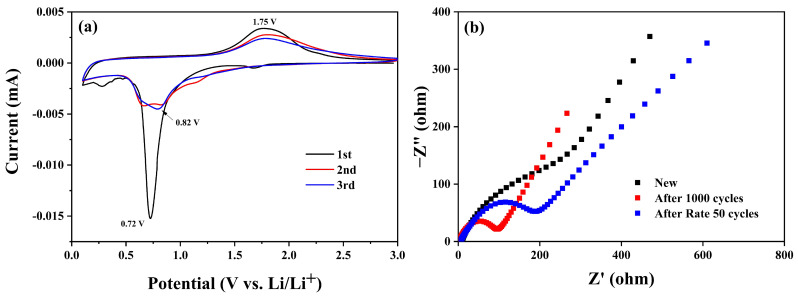
(**a**) Cyclic voltammograms (CV) curves and (**b**) Nyquist plots of NCC–Fe_2_O_3_ composite before and after 1000 cycles and after 50 rate cycles.

To further evaluate the detailed lithium storage process of the NCC–Fe_2_O_3_ composite when used as the electrode of LIBs, cyclic voltammetry (CV) was investigated, and the results are shown in Figure 8a. The CV curve for the initial cycle displays a dominant and sharp peak around 0.72 V, which is assigned to the reduction reaction of Fe^2+^ and Fe^3+^ to Fe^0^ and the formation of the SEI layer at the electrolyte/electrode interface, which is a common phenomenon in transition metal oxides electrodes. A minor peak around 1.7 V is presented for the transformation of Fe_2_O_3_ to the Li_x_Fe_2_O_3_ phase [20,30,54]. During the first anodic scan, a broad peak was observed at about 1.75 V, corresponding to the reversible oxidation of Fe^0^ to Fe^2+^ which is further oxidized to Fe^3+^ (Li_2_Fe_2_O_3_ + 4Li^+^ + 4e^−^ ⬄ 2Fe^0^ + 3Li_2_O) [20,30,55,56]. The appearance of the peak at 0.72 is consistent with the long plateau, which can be found in the galvanostatic charge and discharge profile of the NCC–Fe_2_O_3_ electrode. In the second cycle, the cathodic peaks exhibit different characteristic features in comparison with the first cycle, demonstrating the structural modification of materials. The minor peak at 1.7 V disappeared, and two broad peaks were observed between 0.68 and 0.85 V with a significant decrease in intensity, assigned to the presence of irreversible reactions and structural changes during the first cycle. Furthermore, the anodic peak shows a stable shape and little decay, indicating the good electrochemical reversibility of the electrode. Afterwards, the curve shows a slight shift toward a cathodic peak at 0.82 V and the broadening of its shape, decrease in its intensity, and high remaining trend of the anodic peak, demonstrating the high reversibility and capacity stability of the NCC–Fe_2_O_3_ composite during the delithiation process. The almost complete coincidence of the second and third cycle curves suggests the good cycling stability of the NCC–Fe_2_O_3_ composite, which again confirms the better cycling performance of LIBs (Figure 6a).

Electrochemical impedance spectroscopy (EIS) was also utilized to explain the electrochemical kinetics of the NCC–Fe_2_O_3_ composite electrode and the Nyquist diagram of the electrode before and after 1000 cycles and after 50 rate cycles, with the results shown in Figure 8b. As depicted in Figure 8b, the EIS curves of both electrodes after the rate and cycling process exhibit distinct semicircles in the high-frequency region and oblique straight lines in the low-frequency region, while the new electrode curve does not show a similar shape. The diameter of the semicircles in the high-frequency region is directly proportional to the charge transfer resistance (R_ct_) at the electrolyte–electrode interface. The semicircle diameters of the electrode after 1000 cycles and 50rate cycles are smaller than the new electrode, demonstrating the improvement of electrode electrochemical performance and explaining the increase in specific capacities during cycling (Figure 6). In addition, the specific R_ct_ value of NCC–Fe_2_O_3_ after 1000 cycles (52.8 Ω) is smaller than after 50 rate cycle (103.29 Ω), indicating the better reaction kinetics at the electrode surface when the working cycles increase, and this result agrees with the electrochemical performance shown in Figure 6 and Figure 7.

## 4. Conclusions

In summary, we have successfully fabricated an NCC–Fe_2_O_3_ composite through a simple thermal annealing process. In the composite, NCC has been utilized as a flexible framework to incorporate Fe_2_O_3_ particles via in situ thermal decomposition, to play a supporting role in preventing the aggregation and volume expansion of Fe_2_O_3_. Furthermore, the abundant functional groups on the NCC surface allow it to coordinate with Fe_2_O_3_ particles, while the porous network and large surface enable the homogeneous dispersion of active materials. The prepared NCC–Fe_2_O_3_ composite is greatly improved with respect to electrochemical performance and structural stability. When applied in LIBs, the synthesized electrode not only displayed an excellent initial capacity of 1044.35 mAh g^−1^ and remained at 576.70 mAh g^−1^ after 1000 cycles at 100 mA g^−1^ but also exhibited a stable cyclability and outstanding rate performance, especially under high current densities. Having a simple synthesis process and good performance, the NCC–Fe_2_O_3_ composite could be a promising electrode material for high-performance LIBs. Furthermore, the continuously increasing trend of charge/discharge capacity after 1000 cycles indicates a promising approach to expanding the research on maximum running time exerted by NCC–Fe_2_O_3_ electrodes. In addition, the good rate performance at high current densities at 5 and 10 A g^−1^ shows the high potential for these electrode materials in the high-current battery industries and electronic devices.

## Figures and Tables

**Table 1:** Atomic contents of Fe, C, and O in the samples via energy dispersive X-ray spectroscopy (EDS).

Sample	C (atom %)	Fe (atom %)	O (atom %)	Weight Percentage (Fe/C)
NCC–Fe_2_O_3_	60.25	14.98	24.77	0.39
Fe_2_O_3_	35.13	27.57	37.30	3.65

**Table 2:** Comparison of cycling performance capacities of NCC–Fe_2_O_3_/Fe_2_O_3_ at 100 mA g^−1^.

Composite	Initial Capacity	Initial Coulombic Efficiency	Cycle Number	Remaining Capacity	Coulombic Efficiency
NCC–Fe_2_O_3_	1044.35 mAh g^−1^	69.00%	1000	576.70 mAh g^−1^	99.77%
Fe_2_O_3_	852.35 mAh g^−1^	30.48%	1000	171.82 mAh g^−1^	99.72%

## Data Availability

No new data were created.

## Supplementary Materials

The following supporting information can be downloaded at: https://www.mdpi.com/article/10.3390/polym16050691/s1, Figure S1: Carboxyl groups on the NCC surface obtained through oxidation of the primary hydroxyl groups at the C6 position.
