# Supplementary material for: Nanocrystalline Cellulose-Supported Iron Oxide Composite Materials for High-Performance Lithium-Ion Batteries
Source: Polymers (Basel). 2024 Mar 2;16(5):691. doi: 10.3390/polym16050691 (PMC10934348; doi:10.3390/polym16050691)
Supplement: Supplementary file 1 [file polymers-16-00691-s001.zip › polymers-2888419-supplementary.pdf]

polymers

Supporting Information for

Nanocrystalline Cellulose-Supported Iron Oxide Composite Materials for High-Performance Lithium-Ion Batteries

Quang Nhat Tran, Chan Ho Park * and Thi Hoa Le *

Department of Chemical and Biological Engineering, Gachon University, 1342 Seongnam-daero, Sujeong-gu, Seongnam-si 13120, Republic of Korea; tran.nhat147@gachon.ac.kr
* Correspondence: chhopark@gachon.ac.kr (C.H.P.); lehoa290792@gachon.ac.kr (T.H.L.)

Figure Caption of the Supporting Information

Figure S1. Carboxyl groups on the NCC surface obtained through oxidation of the primary hydroxyl groups at the C6 position.

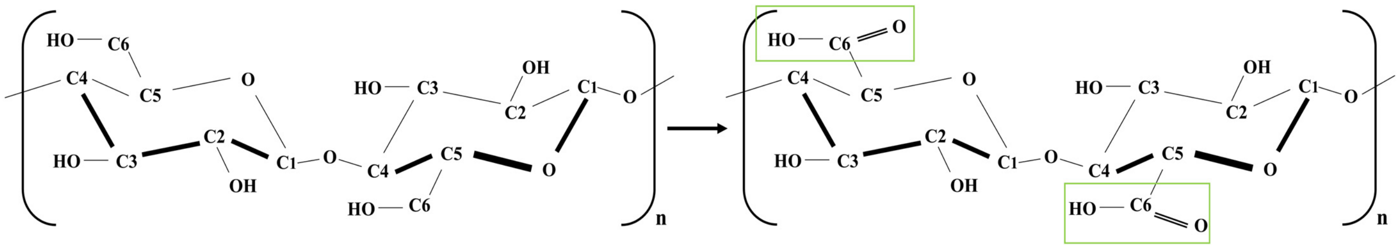

Figure S1. Carboxyl groups on the NCC surface obtained through oxidation of the primary hydroxyl groups at the C6 position.
